# Effectiveness of classification and scoring systems in solitary fibrous tumor prognosis prediction

**DOI:** 10.12669/pjms.42.2.13897

**Published:** 2026-02

**Authors:** Huseyin Fatih Sezer, Aykut Elicora, Salih Topcu, Busra Yaprak Bayrak

**Affiliations:** 1 Huseyin Fatih Sezer Department of Thoracic Surgery, Kocaeli University Medical Faculty, Kocaeli, Turkey; 2 Aykut Elicora Department of Thoracic Surgery, Kocaeli University Medical Faculty, Kocaeli, Turkey; 3 Salih Topcu Department of Thoracic Surgery, Kocaeli University Medical Faculty, Kocaeli, Turkey; 4 Busra Yaprak Bayrak Department of Pathology, Kocaeli University Medical Faculty, Kocaeli, Turkey

**Keywords:** De Perrot classification, Diebold score, Demicco score, England criteria, Fibrous Tumor, Solitary Tapias score

## Abstract

**Objectives:**

Because thoracic solitary fibrous tumors (SFTs) are rare, there is limited information on their clinical features, treatment, and follow-up. The literature mainly comprises individual case reports. Our study aimed to analyse SFT data to identify prognosis-related factors and to compare the performance of two describing approaches and three risk stratification models.

**Methodology:**

We conducted a retrospective review of medical data from 37 patients, including 32 patients with SFT aged 18 years and older who underwent surgical treatment and five patients diagnosed by biopsy at Kocaeli University Hospital between January 2004 and December 2024. Parameters affecting recurrence and survival were investigated, and the effectiveness of existing classification and scoring systems was compared.

**Results:**

Factors potentially associated with recurrence included four or more mitoses (10 HPF) (p=0.023), necrosis (p=0.007), at least 10% Ki-67 positivity (p=0.012), malignancy (p=0.046), and tumor size (p=0.022). Relapses were observed only in the malignant group according to the England classification (p=0.046), only in stage-3 according to the de Perrot classification (p=0.161), only in those with scores of three or higher according to the Tapias classification (p=0.036), only in those with scores of two or higher according to the Diebold classification (p=0.021), and all in high-stage groups (p=0.001) according to the Demicco classification. All recurrences occurred at significant or high scores.

**Conclusions:**

The Tapias, Diebold, and Demicco scoring systems are highly effective in predicting recurrence and prognosis. However, the modified Demicco system is more advantageous and offers a higher level of assessment.

## INTRODUCTION

Thoracic solitary fibrous tumors (SFTs) are rare, and their usual location is the pleura.^1^-^4^ Until 2005, 800-900 cases were reported in the literature, and today, around 1000 cases are documented.^2^,^5^-^7^ Its incidence has been reported as 2.8/100000.^2^,^8^ The exact aetiology is unknown.^7^,^9^,^10^ It was previously regarded as a form of mesothelioma, but later, it was recognised as a distinct entity tumor.^2^,^5^,^11^ These processes have led to confusion concerning clinical and pathological aspects issues.^12^ Furthermore, because tumors are rare, information about their clinical presentation, treatment, and follow-up procedures is limited. The literature generally consists of individual case reports. In fact, there are few studies that include the results of more than 50 patients who were surgically treated, and these are usually multicenter.

Although they generally have a good prognosis, a small percentage (10-30%) may demonstrate malignant behavior. It is clinically important to predict which ones might display malignancy behaviour.^5^,^12^,^13^ To understand this situation, various definitions, classifications, and scoring systems have been established over time.^3^ England defined malignancy criteria based on histopathology, and de Perrot believed that histopathology alone could not reflect prognosis, incorporating morphological appearance into their criteria alongside histological factors features.^5^,^13^,^14^ Later, Tapias, Diebold, and Demicco introduced prognosis prediction using their own scoring systems. Current studies are ongoing to predict the behavior of this tumour group.^15^ These classification and scoring systems were developed based on data from a small number of patients. Several studies are now examining the effectiveness of these approaches. In these studies, classification or scoring systems were generally compared in pairs, but a study in which all of them were compared or their results were analyzed has not yet been presented.

In our study, we aimed to compare the effectiveness of all existing classifications and scoring systems related to this disease, for which limited clinical information is available, using our long-term results, and to identify the factors that may influence a poor prognosis.

## METHODOLOGY

Medical data from 37 patients, including 32 patients with SFTs aged 18 years and older who underwent surgical treatment and five patients diagnosed via biopsy between 2004 and 2024 at Kocaeli University Thoracic Surgery Clinic, were analyzed retrospectively. Seven patients who had surgery were excluded due to incomplete follow-up data, resulting in a final sample of 30 patients.

The patient’s history was reviewed prior to the operation. Physical examination, routine blood tests, pulmonary function tests, and cardiac and respiratory assessments were performed. Radiological examinations, including X-ray and CT scans, were carried out on all patients, and nuclear medicine PET-CT (FDG) was performed on some. Preoperative diagnosis was confirmed through CT-guided tru-cut biopsy in certain cases. Patients who could not be diagnosed beforehand were diagnosed during surgery. The procedures included posterolateral thoracotomy or video-assisted thoracoscopic surgery (VATS). Early follow-up occurred in the outpatient clinic at the 1st, 3rd, and 7th weeks after discharge. Chemotherapy (KT) and radiotherapy (RT) were administered to patients with microscopic residual disease or recurrence, with closer follow-up in collaboration with the oncology department. Long-term follow-up was conducted every six months for the first two years, then annually thereafter.

The malignant criteria for SFTs were established by England et al.^14^ A classification that includes both morphological and histological features was created by De Perrot.^5^ De Perrot et al. classified SFTs as 1- benign tumor with pedicle, 2- benign tumor with broad base, 3- malignant tumor with pedicle, and 4- malignant tumor with broad base.^5^ In our study, benign versus malignant differentiation was made according to England’s and de Perrot’s criteria^5^,^14^, and recurrence risk was estimated based on Tapias et al.^16^, Diebold et al.^17^ and Demicco et al.^18^ scoring systems (Table-I).

**Table-I:** Identification and Classification Systems.

Criterion and Score	*England (1989)*	*de Perrot (2002)*	*Tapias (2013)*	*Diebold (2017)*	*Modified Demicco (2017)*
	Hypercellularity	Hypercellularity	Pleura (Visceral=0, parietal=1)	Mytotic Figures: 4≤ (1)	Age (<55=0, 55≤ =1)
	Mytotic Figures: more than 4	Mytotic Figures: more than 4	Morphology (peducled=0 sessile=1)	Necrosis (1)	Size (<5=0, 5-10=1, 10-15=2,15≤= 3)
	Pleomorfizm	Pleomorfizm	Size (<10= 0, 10≤ =1)	Size (<10=0, 10≤ =1)	Mytotic Figures (0=0, 1-3=1, 4≤ =3)
	Necrosis	Necrosis	Hypercellularity (1)	MIB-1 (< 10%=0, ≥ 10% =1)	Necrosis (<10=0, 10≤ =1)
	Hemorrhage	Hemorrhage Stromal/vascular invasion	Necrosis or Haemorrhage (1)		
			Mytotic Figures: 4≤ (1)		
Significant	1 or more criteria	1 or more criteria	3≤ points	2≤ points	Low: 0-3 points
					Intermedier: 4-5 points
		Stage			High: 6-7 points
		Stage 0: Pedunculated tumor without signs of malignancy^x^			
		Stage 1: Sessile or “inverted” tumor without signs of malignancy^x^			
		Stage 2: Pedunculated tumor with histologic signs of malignancy^x^			
		Stage 3: Sessile or “inverted” tumor with histologic signs of malignancy^x^			
		Stage 4: Multiple synchronous metastatic tumors			

### Ethical considerations:

All procedures involving human subjects were performed in accordance with the ethical standards of the institutional and national research committee, the 1964 Helsinki declaration, and its later amendments. This study adheres to the STROCSS criteria for reporting cohort studies in surgery. Written and verbal informed consent was obtained from all patients.

### Ethical Approval:

It was granted by the Kocaeli University Institutional Ethics Board Review (Approval No: 2024/127; Dated: March 12, 2024).

### Inclusion & Exclusion Criteria:

Patients over 18 years of age diagnosed with solitary fibrous tumour, who have been followed up by our clinic and for whom sufficient data is available, were included in the study. Patients under 18 years of age, with insufficient data or whose follow-up was not conducted at our clinic, were excluded from the study.

### Data collection and analysis:

Patient data were anonymised and accessed through hospital information systems, imaging systems, outpatient clinic follow-up records, and telephone calls. Follow-up, survival, mortality, recurrence, and metastasis durations were calculated using the date of diagnosis or the date of surgery as references. Postoperative mortality was defined as deaths within the first month after the operation. Patient data were anonymised, and demographic characteristics, tumor details, comorbidity information, clinical features, pathological data (including histological, morphological, and immunological aspects), radiological findings, preoperative, intraoperative, and postoperative outcomes, non-surgical treatments, recurrence, metastasis, overall survival (OS), disease-free survival (DFS), follow-up data, and long-term outcomes were analyzed.

### Statistical analysis:

All statistical analyses were performed using IBM SPSS for Windows version 29.0 (IBM Corp., Armonk, NY, USA). Shapiro-Wilk’s test was used to assess the normality assumption. Continuous variables were presented with mean±standard deviation or median and interquartile range (IQR). Categorical variables were summarized as counts and percentages. Comparisons between groups were performed using Mann-Whitney U test since the normality assumption did not hold. Associations between categorical variables were examined using the Chi-square test. Kaplan-Meier method with the log-rank test was used for the survival analysis. A p-value<0.05 was considered statistically significant.

## RESULTS

The mean age was 59.40±13.23 years. The gender distribution was 16 (53.3%) males and 14 (46.7%) females. 20 (66.7%) patients had a smoking history. Pleural effusion was observed in six (20%) patients, all of whom belonged to the malignant group. Preoperative diagnosis was established by percutaneous biopsies in 19 (63.33%) patients. Isolated mass excision was performed in 21 (84%) patients, whereas lung parenchyma was also removed with wedge resection along with the mass in 4 (16%) patients. Incomplete resection was performed in two (8%) patients. Older age (p=0.001), smoking (p=0.03), and pleural effusion (p=0.038) were significantly more common in the malignant group (Table-II). 13 (52%) tumors originated from the parietal pleura and 12 (48%) from the visceral pleura. The median tumor size was 8.75 cm.

**Table-II:** Demographic and Surgical Characteristics.

	Number & Frequency			
	Total	Benign	Malign	p
Age*	59,40±13,23	47,11±11,23	64,67±10,32	0.001
Sex				0.542
Male	16 (53,3%)	4 (44,44%)	12 (57,14%)	
Female	14 (46,7%)	5 (55,56%)	9 (42,86%)	
Smoking	20 (66,7%)	4 (44,44%)	16 (76,19%)	0.03
Side				0.482
Left	16 (60%)	4 (44,44%)	13 (61,9%)	
Right	12 (40%)	5 (55,56%)	8 (38,1%)	
Pleural effusion	6 (20%)	0	6 (100%)	0.038
Preoperative procedures				
Fine-needle aspiration biopsy**	0/2 (0%)	0	0/2 (0%)	
Tru-cut biopsy**	19/19 (100%)	7/7(100%)	12/12(100%)	1.00
Surgical procedures				0.864
Isolated mass excision	21 (84%)	6 (66,67%)	15 (71,43%)	
Wedge Resection	4 (16%)	0	4 (19,05%)	
Only biopsy	5(16,67%)	3 (33,33%)	2 (9,52%)	
Incomplete resection	2 (8%)	0	2 (9,52%)	
* year				

Tumor morphology showed that 6 (24%) tumors were pedunculated and 19 (76%) were sessile. Mitosis of 4 or more (10 HPF) was observed in five (16.7%) patients, necrosis in 16 (53.3%) patients, high cellularity in 6 (24%) patients, and pleomorphism in four (13.3%) patients. CD 34 positivity was seen in 27 (90%) patients, bcl-2 positivity in 29 (96.7%) patients, STAT-6 positivity in nine (90%) patients, and Ki-67 of 10% or more was found in 12 (40%) patients. Although tumor size was numerically larger in the malignant group, this difference was not statistically significant (p=0.150). Pleomorphism was observed only in the malignant group, but it was not statistically significant (p=0.056).

Tumors with sessile morphology predominated in the malignant group (p=0.032). The STAT-6 marker, which was introduced into routine practice after a certain date, was used in 10 patients. Its sensitivity for solitary tumors was high at 90%, but it was not significant in differentiating benign from malignant lesions (p=0.840). When pathological markers and immunohistochemistry were evaluated, only mitosis, necrosis, and high cellularity showed significantly higher incidence in the malignant group (p=0.046, p=0.001, and p=0.038, respectively) (Table-III).

**Table-III:** Pathological and Immunological Features.

	Number & Frequency			
	Total	Benign	Malign	p
Pleural pattern				0.864
Parietal	13 (52%)	4 (44,44%)	9 (42,86%)	
Visceral	12 (48%)	2 (22,22%)	10 (47,62%)	
Tumor size*	8,75	8,5	11	0.150
Morphology				
Peduncle	6 (24%)	3 (33,33%)	3 (14,29%)	
Sesssil	19 (76%)	3 (33,33%)	16 (76,19%)	0.032
***Pathological Markers***				
Mitosis**	5 (16,7%)	0	5 (23,81%)	0.046
Necrosis	16 (53,3%)	0	16 (76,19%)	0.001
High cellularity	6 (24%)	0	6 (28,57%)	0.038
Pleomorphism	4 (13,3%)	0	4 (19,05%)	0.056
CD34	27 (90%)	8 (88.89%)	19 (90.48%)	0.920
bcl-2	29 (96,7%)	8 (88.89%)	21 (100%)	0.960
STAT 6***	9/10^x^	5(100%)	4 (80%)	0.840
ki-67 (more 10%)	12 (40%)	2 (22,22%)	10 (47,6%)	0.371
* cm **4/10 ≤ (HPF)	*** +/All	^x^ +/All performed		

Follow-up was 50±24.01 months. Recurrence was noted in 5 (16.7%) patients, and metastasis to the contralateral lung occurred in 1 (4.76%) patient. Chemotherapy was given to four (13.3%) patients, while radiotherapy was administered to six (20%) patients, all within the malignant group. All recurrences and metastases to the contralateral lung occurred exclusively in the malignant group (p=0.046). During follow-up, a total of 11 (36.7%) patients died. Of these, 10 (11.11%) were from the malignant group (p=0.032), with only one mortality in the benign group, which was a surgical death within the first 30 days. Two (9.52%) of the deaths in the malignant group were related to the disease (SFT) (Table-IV).

**Table-IV:** Follow-up and Prognosis Features.

	Number & Frequency			
	Total	Benign	Malign	p
Follow-up*	50±24,01	52.22±29,75	49.05±21.95	0.940
Recurrence	5 (16,7%)	0	5 (23.81%)	0.046
Metastasis	1(3,3%)	0	1 (4.76%)	−
Chemotherapy	4 (13,3%)	0	4 (19.05%)	0.056
Radiotherapy	*6(20%)*	0	6 (28.57%)	0.038
Death	11 (36,7%)	1 (11.11%)	10 (47.62%)	0.032
In the first 30 days	1(3,3%)	1 (11.11%)	0	−
DOC	8 (26,7%)	0	8 (38.10%)	0.028
DOD	2 (6,7%)	0	2 (9.52%)	−
DOC, dead for other causes; DOD, dead of disease				
* Month				

The median time to recurrence was 36 (22-58) months. Recurrence was observed only in the necrosis, 10%≤ Ki-67 positive, and malignant groups (p=0.007, p=0.012, p=0.046, respectively). Recurrence occurred in three patients with four or more mitoses (10 HPF) (p=0.023) and in two patients with pleomorphism (p=0.23). (Table-V) In the multivariate analysis involving significant parameters, only malignancy remained significant. The median age was higher in the recurrence group (65 vs. 57) (p=0.481). Among the demographic and morphological features in the recurrence group, only size was significant, being larger in the malignant group (16 cm vs. 6 cm) (p=0.022).

**Table-V:** Factors that may be associated with recurrence.

	Recurrence number	Total Recurrences	p
***Pathological and Immunological***			
	Mitosis >4/10 HPF 3	5	0,023
	Mitosis (+) 5	5	
	Necrosis (+) 5	5	0,007
	Pleomorphism (+) 2	5	0,23
	ki-67 (≥10%) (+) 5	5	0,012
	Malign (+) 5	5	0,046
	Mitosis >4/10 HPF 3		
***Demographic and Morphological***			
	Age		0,481
	Size		0,022

**Fig.1:**
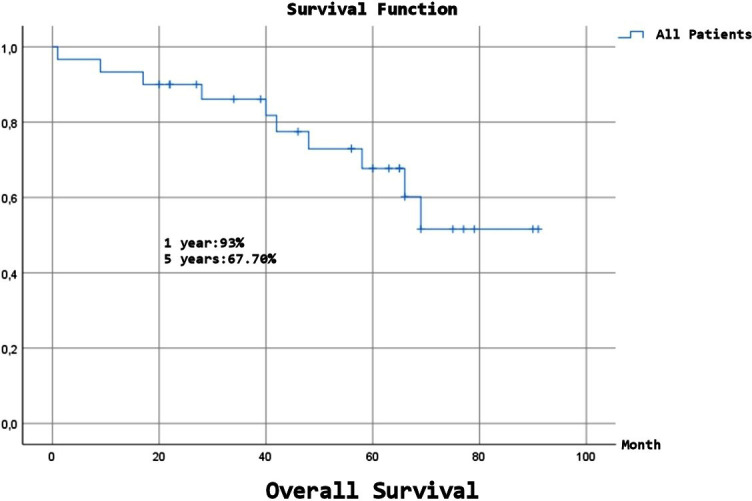
Over all survival (all patients).

**Fig.2:**
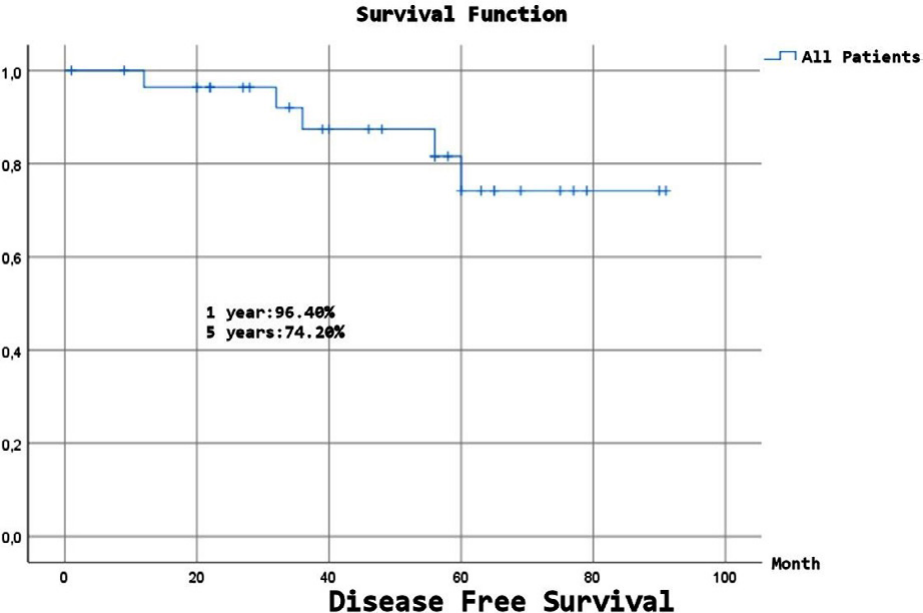
Disease free survival (all patients).

**Fig.3:**
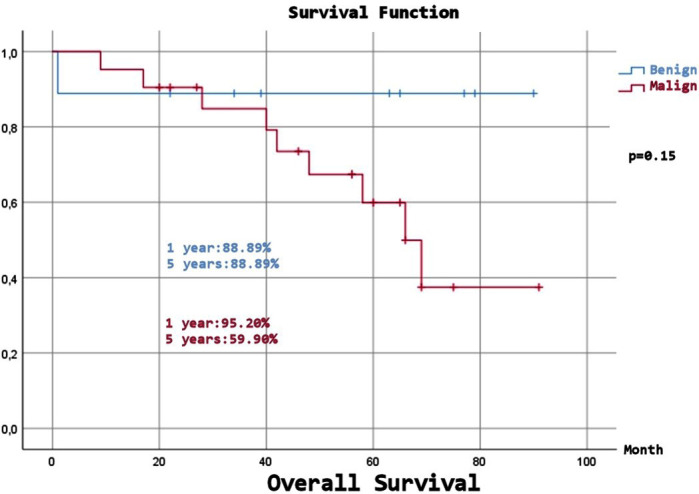
Overall survival (benign and malign patients).

**Fig.4:**
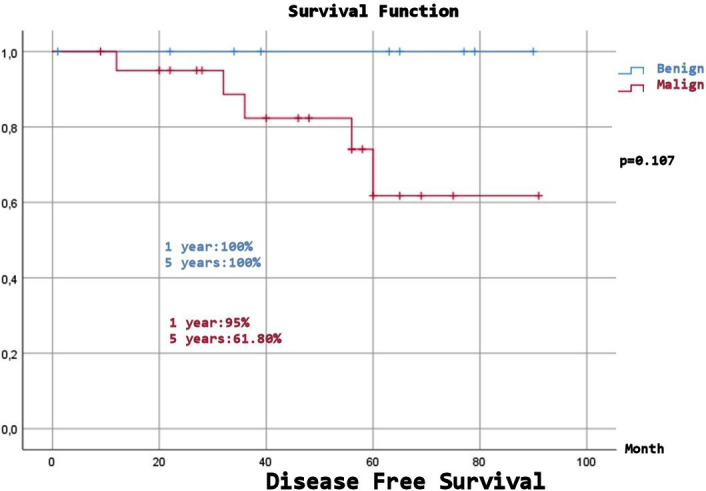
Disease free survival (benign and malign patients).

Expected Overall Survival (OS) was 68.58±5.67 months, and although a numerical difference was observed, there was no statistically significant difference between the benign and malignant groups (p=0.15). Expected Disease-Free Survival (DFS) was 78.72±4.89 months, and despite a numerical difference, there was no statistically significant difference between the benign and malignant groups (p=0.107). (Fig.1-4), (Table-VI).

**Table-VI:** Long-Term Survival and Disease-Free Times.

		DFS	OS
All	1 year	96,40%	93%
	2 year	96,40%	90,00%
	5 year	74,20%	67,70%
	10 year	74,20%	51,60%
***Benign***			
	1 year	100%	88,89%
	2 year	100%	88,89%
	5 year	100%	88,89%
	10 year	100%	88,89%
***Malign***			
	1 year	95%	95,20%
	2 year	95%	90,50%
	5 year	61,80%	59,90%
	10 year	61,80%	37,40%
			
	p=0.107		p=0.15
OS: Overall Survival			
DFS: Disease Free Survival			

Relapses were observed only in the malignant group according to the England classification (p=0.046), only in stage-3 according to the de Perrot classification (p=0.161), only in patients with scores of three or higher according to the Tapias classification (p=0.036), only in those with scores of two or higher according to the Diebold classification (p=0.021), and all occurred in high-stage cases (p=0.001) according to the Demicco classification. All relapses were associated with significant or high scores. (Table-VII) Due to the low number of recurrences, multi-variable analysis could not be performed to compare the classifications.

**Table-VII:** Recurrence Prediction Performances of Classification Systems.

Classification	n (%)	Recurrence
***England Criterion***		
Benign	9 (30%)	
Malign	21 (70%)	all /(p=0.046)
***de Perrot***		
0	5 (16,7%)	
1	3 (10%)	
2	1 (3,3%)	
3	16 (53,3%)	all /(p=0,161)
4	0	
***Tapias***		
3≤ points	12 (48%)	all /(p=0,036)
3 > points	13 (52%)	
***Diebold***		
2≤ points	13 (43,3%)	all /(p=0,021)
2 > points	17 (56,7%)	
***Modified Demicco***		
Low:	15 (50%)	
Intermedier:	7 (23,3%)	
High:	8 (26,7%)	all /(p=0,001)

n: number.

## DISCUSSION

In our study of these tumours, which are rarely observed and have limited literature, the factors directly affecting prognosis are malignancy, increased mitotic count, presence of necrosis, recurrence, and Ki-67 ≥ 10%. Additionally, advanced age, large size, and pleomorphism are indirect factors. Although the Tapias, Diebold, and Demicco scoring systems are quite effective in predicting recurrence and prognosis, the modified Demicco system is more practical and provides a higher level of assessment.

Most SFTs present as benign behaviour.^5^,^6^,^19^ However, there have also been cases of benign to malignant transformation reported.^5^ Therefore, predicting tumors that may exhibit malignant behavior is important in clinical practice. There are few studies in the literature on SFTs, including results from 50 or more patients who have undergone surgery, and these are generally multicenter. Consequently, more case series are needed to better analyses disease behavior, follow-up, and treatment approaches. Our study aimed to identify factors affecting prognosis by analyzing data on SFTs and to compare the performance of two grouping methods and three risk classification models proposed in the literature regarding prognosis and long-term follow-up results.

The reported rate of malignancy in SFTs is 12-37%.^1^,^5^,^20^ Benign to malignant conversion has been reported in cases and is associated with size.^5^,^21^,^22^ Malignant tumors are expected to be larger, more symptomatic, atypically localized, younger in age, sessile, and multifocal.^1^,^4^,^6^,^7^,^22^-^26^ In our study, age was significantly higher in malignant patients (65-42) (p=0.001). Tumor size was numerically larger in malignant patients (11-8.5 cm) (p=0.150), and most of those with ki-67 (10% and above) + were in the malignant group (p=0.371).

Recurrences are observed more often in the first 2-3 years.^1^,^2^,^25^ Recurrence rates range from 0 to 17% in benign cases and from 13.6 to 66.7% in malignant ones cases.^2^,^3^,^6^,^13^,^16^,^17^,^22^,^24^,^26^-^28^ Sesile morphological type, resections larger than the wedge, and CD34 negativity are significant indicators of malignancy recurrence.^25^,^26^ In our study cohort, 16.7% of recurrences occurred in patients with malignancies, with a median recurrence time of 36 months. Although no recurrence was observed in patients without mitosis, recurrences were seen in three patients with a mitosis count of four (10 HPF) or more and in two patients with a mitosis count of four (10 HPF) (p=0.023). Recurrences were only recorded in patients with necrosis (p=0.007), malignancy (p=0.046), and larger tumor size (p=0.022). Recurrence was more significant (p=0.012) in patients with ki-67 positivity ≥10%. Although the mean age of patients with recurrence was higher (65 vs. 57), the difference was not statistically significant (p=0.481). However, since malignancy is a major factor in recurrence and the mean age of malignant patients was higher (p=0.001), age has an indirect significance regarding recurrence. No correlation was found between pleomorphism and recurrence (p=0.230). Nonetheless, it was hypothesised that a significant correlation might exist because of the increase in cases, as these were observed exclusively in the malignant group.

Malignancy factors include originating from the parietal pleura or chest wall, isolated resection, malignant pleural effusion, large size (10 cm), mitotic number > 10 (10 HPF), and a high proliferation rate.^6^,^8^,^13^ The five and ten years DFS rates are reported to be 82-95% and 67-90.8%, respectively, and this rate is 58-72.1% to 60.5% in the malignant group, and 95.7-100% in the benign group (five years only).^4^,^6^,^8^,^13^,^16^,^23^,^25^ In our study, DFS was 96.4% at two years, 74.2% at five years, and 74.2% at 10 years. Since there was no recurrence in the benign group, it was 100%, while in the malignant group, it was 95% at two years, 61.8% at five years, and 61.8% at ten years (p=0.107). Our findings show DFS rates align with the literature. There was a numerical difference between the benign and malignant groups, and we expect a statistically significant difference as the number of cases increases.

Studies have reported five and ten years survival rates of 86-94% and 72.1-77%, respectively, with a median survival of 14-24 years.^3^,^9^,^10^,^16^,^23^,^25^ In benign SFT cases, five years survival rates are 89-100%; in malignant cases, five years survival rates range from 45.5% to 89%, and ten years survival rates are 66.9%.^3^,^6^,^23^-^25^,^28^ We attribute the numerical differences between the results of the studies to variations in the number of malignant patients and surgeries. In our study, OS was 90% at two years, 67.7% at five years, and 51.6% at ten years. OS in the benign group was 88.89% at all follow-up time points, whereas OS in the malignant group was 90.5% at two years, 59.9% at five years, and 37.4% at ten years. The number of patients with malignancies was significantly higher in our patient population. Therefore, although our OS rate was slightly lower for all patients compared with that in the literature, the rate in patients with malignancies was comparable. The expected OS was 80.11 months in benign patients and 64.01 months in malignant cases (p=0.15). We believe that the statistical difference between the two groups will become more evident as the number of cases increases.

In the study by Tan et al.^26^, OS was 76% and DFS was 53% in malignant patients. When the survival graph from this study is analyzed, it is observed that both rates are nearly equal during the first two years, with the disparity emerging after the 3rd year and then progressing in tandem with these rates. Similarly, in our study of malignant tumors, the difference widened after the 2nd year.

Factors affecting prognosis have been reported to include incomplete resection, recurrence, large size (10 cm), and pedicled genetic molecular factors encapsulated.^4^,^6^,^16^,^17^,^24^,^26^,^29^ On the other hand, some authors have reported that tumor size, the pleura from which the tumor originates, or being symptomatic are not effective in prognosis.^5^,^6^,^8^,^13^ The most important prognostic factor related to surgical treatment is complete blockage removal resection.^20^ In the study by Lahon et al.^25^, which included multifactorial analysis, it was found that factors related to short-term survival in malignant tumors were vena cava compression, multifocality, resections larger than wedge, and recurrence, with recurrence being the most significant factor. In our study, the factors directly influencing prognosis were recurrence, malignancy, ki-67 > 10%, mitotic count of 4 (10 HPF) or more, necrosis; while the indirect factors were age, tumor size, and pleomorphism. Due to numerical limitations, we could not compare these factors through multifactorial analysis; however, the most impactful factor was recurrence based on individual assessments.

Surgery and chemoradiotherapy are employed in treating SFT. The timing of radiotherapy remains a contentious issue. Haas et al.^30^ demonstrated that surgery combined with radiotherapy offers no significant advantage in overall survival (OS), although they recommend it for tumors with poor resection margins or high mitotic activity. Radiotherapy can influence surgical outcomes and potentially increase complication rates due to its effects on tissue. In our view, radiotherapy is a viable option following R1 resections, in cases of unresectable tumors, or with metastases.

Since anatomical classification systems cannot be used effectively, specialized classifications that are not based on large patient groups are employed to assess prognosis and recurrence.^3^ The low incidence of SFT may be the reason. Apart from the definitions of malignancy, the authors developed their own scoring systems beyond malignancy criteria, incorporating factors that may influence prognosis and recurrence. They occasionally compared their scoring systems with others. New scoring studies are ongoing progress.^13^,^17^ While some authors have reported that isolated histological or morphological features can be useful for prediction, others have found that these alone are not enough. For example, in a study by Tan et al., recurrence rates were reported as 2%, 8%, 14%, and 63% according to Perrot’s classification.^26^

However, Tapias et al. found no significant difference in these parameters when comparing high- and low-risk groups according to Perrot and England’s scoring.^16^ In this study, the performance of these definitions, classifications, and systems was compared. All recurrences were observed in England’s malignant group (p=0.046), de Perrot stage 3 (p=0.161), Tapias’ cases with a total score of three and above (p=0.036), Diebold’s cases with a total score of two and above (p=0.021), Demicco’s stage-3 (p=0.001).^5^,^14^-^18^ Based on these results, it was observed that England’s criteria should be used for malignancy, de Perrot’s classification was partially effective, and the Tapias, Diebold, and Demicco scoring systems were very successful in predicting recurrence. Given our current number of cases, we consider it premature to present a statistical comparison of the superiority of any one scoring system. We plan to include this analysis in future studies with a larger sample size. However, if we must comment, the modified Demicco classification was considered to be slightly superior to the other scoring systems because it assesses more variables, creates more groupings, and most of the recurrences in our study occurred in the highest scoring stage.

Our study contributes to the literature as a case series, unlike publications that mostly consist of isolated case reports on this very rare tumor. Being a single center can sometimes be a disadvantage, but it has also allowed us to closely follow up with the patients. Our study has compared the current classifications with each other and also presented our own experiences. Therefore, our study will be a reference for future researchers to compare with.

### Limitations:

The weaknesses of our study included its retrospective, single center design and relatively small number of cases. We faced the challenge of studying a rare tumor, which prevented us from detecting a statistical difference in some instances where there was a numerical difference. Nevertheless, we gathered valuable data by comparing the effectiveness of all classifications used to predict SFT prognosis and behavior.

## CONCLUSION

As a result, the factors directly affecting prognosis are malignancy, a mitotic count of 4 (10 HPF) or more, necrosis, recurrence, and ki-67 ≥ 10%, while the factors indirectly influencing prognosis include increasing age, enlarging size, and pleomorphism. For malignancy criteria, England’s standards should be applied, and de Perrot’s classification is partly functional. The Tapias, Diebold, and Demicco scoring systems are highly effective in predicting recurrence and prognosis. However, the modified Demicco system is more useful and offers a higher level of evaluation. Studies with larger patient populations will help determine which scoring system is most effective, but this is likely to take a long time due to the rarity of the disease.

### Authors’ Contribution:

**HFS:** Conceived, designed and did statistical analysis & editing of manuscript, is responsible for integrity of research.

**HFS, AE, ST & BYB:** Did data collection and manuscript writing.

**HFS:** All authors have read and approved the final manuscript.
